# Working well: a systematic scoping review of the Indigenous primary healthcare workforce development literature

**DOI:** 10.1186/s12913-019-4580-5

**Published:** 2019-10-29

**Authors:** Janya McCalman, Sandra Campbell, Crystal Jongen, Erika Langham, Kingsley Pearson, Ruth Fagan, Ann Martin-Sardesai, Roxanne Bainbridge

**Affiliations:** Central Queensland University, QLD, Cairns, Australia

**Keywords:** Human resource management, Personnel management, Workforce planning, Retention, Recruitment, Personnel selection, Health personnel, Professional development, Capacity development, Skills development

## Abstract

**Background:**

Strong and effective workforce models are essential for improving comprehensive Indigenous primary healthcare service (PHC) provision to Indigenous peoples in Canada, Australia, New Zealand and the USA (CANZUS nations). This review systematically scoped the literature for studies that described or evaluated models and systems that support the sustainability, capacity or growth of the Indigenous PHC workforce to provide effective PHC provision.

**Methods:**

Eleven databases, 10 websites and clearinghouses, and the reference lists of 5 review articles were searched for relevant studies from CANZUS nations published in English from 2000 to 2017. A process of thematic analysis was utilised to identify key conditions, strategies and outcomes of Indigenous PHC workforce development reported in the literature.

**Results:**

Overall, 28 studies were found. Studies reported enabling conditions for workforce development as government funding and appropriate regulation, support and advocacy by professional organisations; community engagement; PHC leadership, supervision and support; and practitioner Indigeneity, motivation, power equality and wellbeing. Strategies focused on enhancing recruitment and retention; strengthening roles, capacity and teamwork; and improving supervision, mentoring and support. Only 12/28 studies were evaluations, and these studies were generally of weak quality. These studies reported impacts of improved workforce sustainability, workforce capacity, resourcing/growth and healthcare performance improvements.

**Conclusions:**

PHCs can strengthen their workforce models by bringing together healthcare providers to consider how these strategies and enabling conditions can be improved to meet the healthcare and health needs of the local community. Improvement is also needed in the quality of evidence relating to particular strategies to guide practice.

## Background

A strong and effective workforce is needed to underpin comprehensive primary healthcare efforts by primary healthcare services (PHCs). Primary healthcare is important because it focuses on healthcare throughout the lifespan and can deliver better health outcomes, efficiency and improved quality of care compared to other models [1]. Globally, particularly in rural and remote areas, PHCs face challenges in defining and operationalising an optimal workforce model that responds to the needs for primary healthcare delivery [2, 3]. Such a workforce requires stability, leadership, role clarity, support and coordination [2, 4]. PHCs and the workforce models that underpin them have traditionally been framed mainly to address acute conditions, yet they are also faced with a high and increasing burden of chronic disease in the populations they serve [5, 6]. Addressing chronic disease and wellbeing creates a greater demand for patient-centred care, community-based health services, and personalized long-term care [3]. Health workforce strategies therefore increasingly need to incorporate health promotion, prevention, treatment, rehabilitation and palliative care services, and to work through team-based care [3].

Workforce development clearly requires multifaceted strategies, but there is no one size fits all option [2, 7]. International studies have suggested that attention to workforce issues such as leadership, motivation and support can make or break efforts to improve healthcare delivery. One study theorised five key workforce development strategies: 1) recruiting staff with skills in service transformation; 2) redesigning and creating new roles; 3) enhancing workforce planning; 4) linking staff development to service needs; and 5) creating opportunities for shared learning and knowledge exchange [8]. This review examines the literature from Canada, Australia, New Zealand and the United States (CANZUS nations) on workforce models and systems that support the effectiveness, sustainability and/or growth of Indigenous PHCs. The four CANZUS nations share a history of British colonisation as an underlying determinant of health, and despite having high rankings on the United Nations Development Programme’s *Human Development Index,* have produced inconsistent results in Indigenous health and well-being improvement over time and relative to their non-Indigenous populations [9, 10]. We use the United Nations definition of Indigenous peoples, that is: “the descendants of those who inhabited a country or a geographical region at the time when people of different cultures or ethnic origins arrived… and later became dominant through conquest, occupation, settlement or other means” [11]. The term workforce is used to describe the people engaged in or available for work in an Indigenous PHC organisation. We suggest that through Indigenous PHCs, workforce development is an important contributing factor to improving healthcare outcomes for Indigenous people in the CANZUS nations [10].

The patterns of Indigenous health in the CANZUS nations can be explained by the social determinants of health, that is, the aspects of birth, growth, education, living, and working; use of healthcare services; and structural factors such as socioeconomic policy that shape the conditions of daily living [10, 12, 13]. In some rural and remote areas in which Canadian and Australian Indigenous people live, however, poor infrastructure, low population density, and migratory patterns make it difficult to access high-quality essential health services, medicines, and vaccines [10]. There are also access issues for Indigenous people for health and prevention services (such as primary care programmes, vaccination programmes, antenatal care, chronic diseases management, mental health services, and cancer services) [10]. In Australia, for example, the burden of chronic diseases comprises 70% of the health disparity between Indigenous and other Australians, making clear the need to respond better to chronic disease [14]. However, PHCs operate at a cultural interface [15] between Western medical and Indigenous ways of being, knowing and doing, and there is added complexity in developing and implementing workforce improvement efforts in Indigenous PHC.

Health workforce strategies have been developed, such as training Indigenous health professionals, cross-cultural competence in professional and patient relationships, incorporating traditional Indigenous health practices and practitioners into primary health care, and promoting knowledge of Western and Indigenous systems [10, 16, 17]. In some countries there is also an emerging focus on wellness-based approaches, family centred models of care, and Indigenous community-controlled management of primary health-care services [18–20]. Indigenous frameworks, such as the national Australian Indigenous health workforce development framework, focus particularly on supporting the pipeline of Indigenous graduates into health professions, supporting their recruitment, retention, skills and capacity; and providing culturally safe and responsive workplace environments through workforce planning [21].

Several reviews of the Indigenous PHC workforce literature describe conditions and strategies that influence strategic human resource management efforts that in turn, aim to achieve improved healthcare performance [4, 22, 23]. Government policies determine the availability of resourcing for workforce development efforts, but disciplinary silos and restrictions imposed by complex funding streams and governance models create challenges in developing consistent, integrated workforce models [2, 7, 21]. ‘System wide shortages’ of healthcare professionals are apparent, particularly in regional and remote locations (e.g. [24]). Reviews from Australia and the U.S.A. found that Indigenous health practitioners are often underrepresented and underutilised [4, 25]. Training pathways, qualifications and efforts to improve inclusiveness and cultural safety are also needed [4, 25]. For non-Indigenous professionals, longevity required clinical experience and access to professional development; supervision and peer support; and cultural competence and perceived connectedness with the community in which they were located [4]. Strengthening workforce capacity requires tailoring broad approaches to local need; identifying what works, for whom, and why [26].

This paper reviews the Indigenous PHC workforce literature to identify what is known about the development and/or implementation of workforce models in Indigenous PHCs in Canada, Australia, New Zealand and the United States, and identify the evidence gaps. The research question was: What workforce models and systems support effectiveness, sustainabilty and/or growth of Indigenous PHCs? The objectives were to: 1) report on the quantity and nature of available literature; and 2) identify the enabling conditions, strategies, challenges and impacts of implementing workforce models for Indigenous PHC. The review was positioned specifically to inform the efforts of one Australian Indigenous PHC for enhancement of their health workforce environment and systems. Following its transition of governance to community control, the management team of Gurriny Yealamucka Health Service aimed to implement workforce enhancements to further strengthen the workforce and provide a model of healthcare focused on early intervention and health education. The issues entailed are common to many Indigenous PHCs [21].

## Methods

A written protocol for the systematic scoping review was developed and circulated to Gurriny and the research team to ensure that the review results were fit for purpose and that there was consensus on the parameters of the proposed review, the definitions of terms, methods for the search, screening, extraction of data and analysis and synthesis of the literature. It entailed systematically searching, selecting and synthesizing existing knowledge to map key concepts, types of evidence, and gaps in research [27].

### The search strategy - inclusion/exclusion criteria

**Fig. 1:**
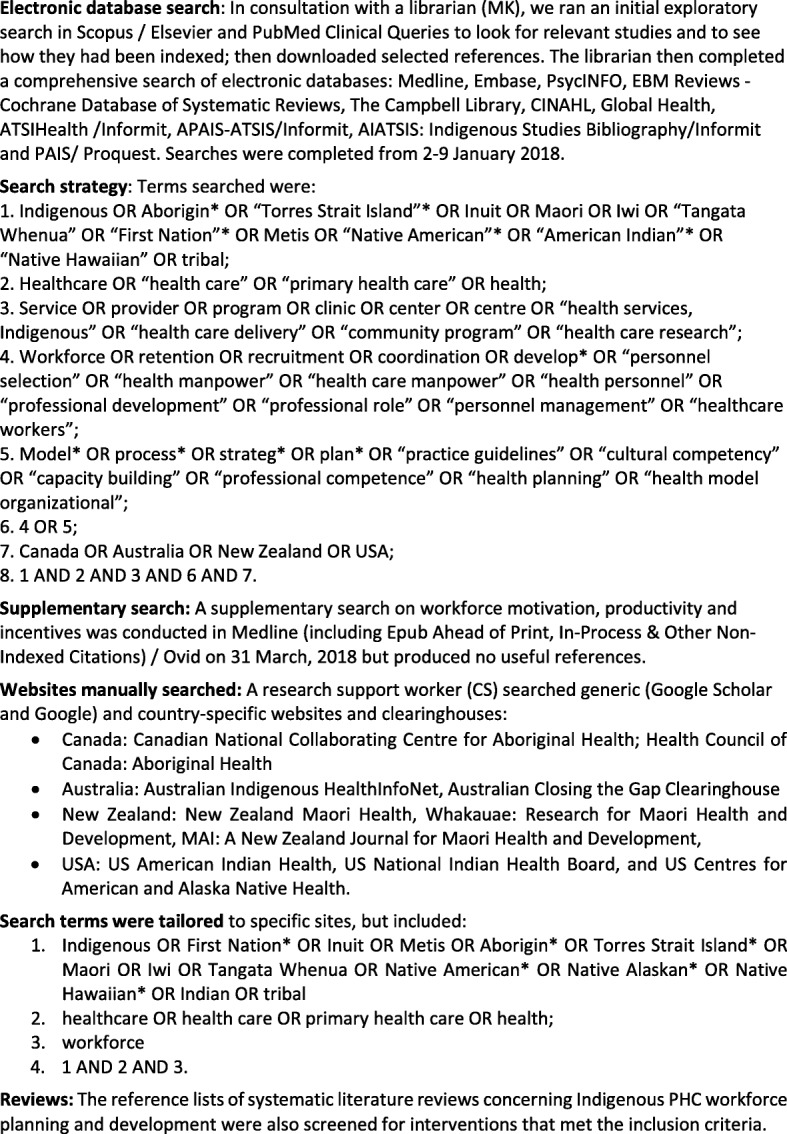
Search strategy

The search strategy is outlined in Fig. 1.

Studies were included if they were:

1. From Canada, Australia, New Zealand and the United States;
2. Published in English, peer reviewed and grey literature if electronically available;
3. Published between January 2000 and December 2017 (the start date was consistent with identification by the World Health Organisation that the health workforce is the most important of all health system inputs [28];
4. Concerned with Indigenous populations;
5. Focused on Indigenous primary healthcare (e.g. not student education/ training); and
6. Aimed to describe or evaluate workforce models that have been developed, implemented or tested in Indigenous PHC (e.g. Indigenous and non-Indigenous employment strategies and roles, professional development opportunities, productivity/ incentive strategies, career advancement pathways and retention strategies).

The Medline search strategy is provided as an (See Additional file 1: Table S1).

### Study identification and selection

The combined database searches were imported into a bibliographic citation management software, EndNote X8. The titles and abstracts were screened respectively by co-authors (SC and JM) to remove articles that were irrelevant to the review. A detailed inclusion/exclusion criterion assisted in the full-text assessment of the remaining publications, which was conducted by two blinded screeners (JM, EL). Resulting disagreements were resolved by discussion until 100% agreement was achieved.

### Study design/quality

The focus of our research question on the characteristics of workforce models meant that it was inappropriate to use a traditional “hierarchy of evidence” to measure the quality of studies. Hierarchies of evidence that place randomised controlled trials as the highest level of evidence and other methods below are used to measure what works, and have increasingly been critiqued as unhelpful if used to answer other questions or varied types of research methodologies [29]. In this review, studies were generally qualitative, using emerging and contextual empirical methodologies. We also included expert commentaries and concept papers since they were considered likely to have crucial lessons for PHCs decision makers regarding expected and unexpected effects [29]. Furthermore, in Indigenous health research, the very use of the term evidence has been critiqued as privileging a one size fits all and Eurocentric approach, with some Indigenous researchers stating a preference for the term “wise practices” that are “reflective of Indigenous peoples’ worldviews and ways of creating knowledge” [30]. However, there are not yet Indigenous frameworks available for evaluating the quality or extent to which practices are “wise”.

**Fig. 2:**
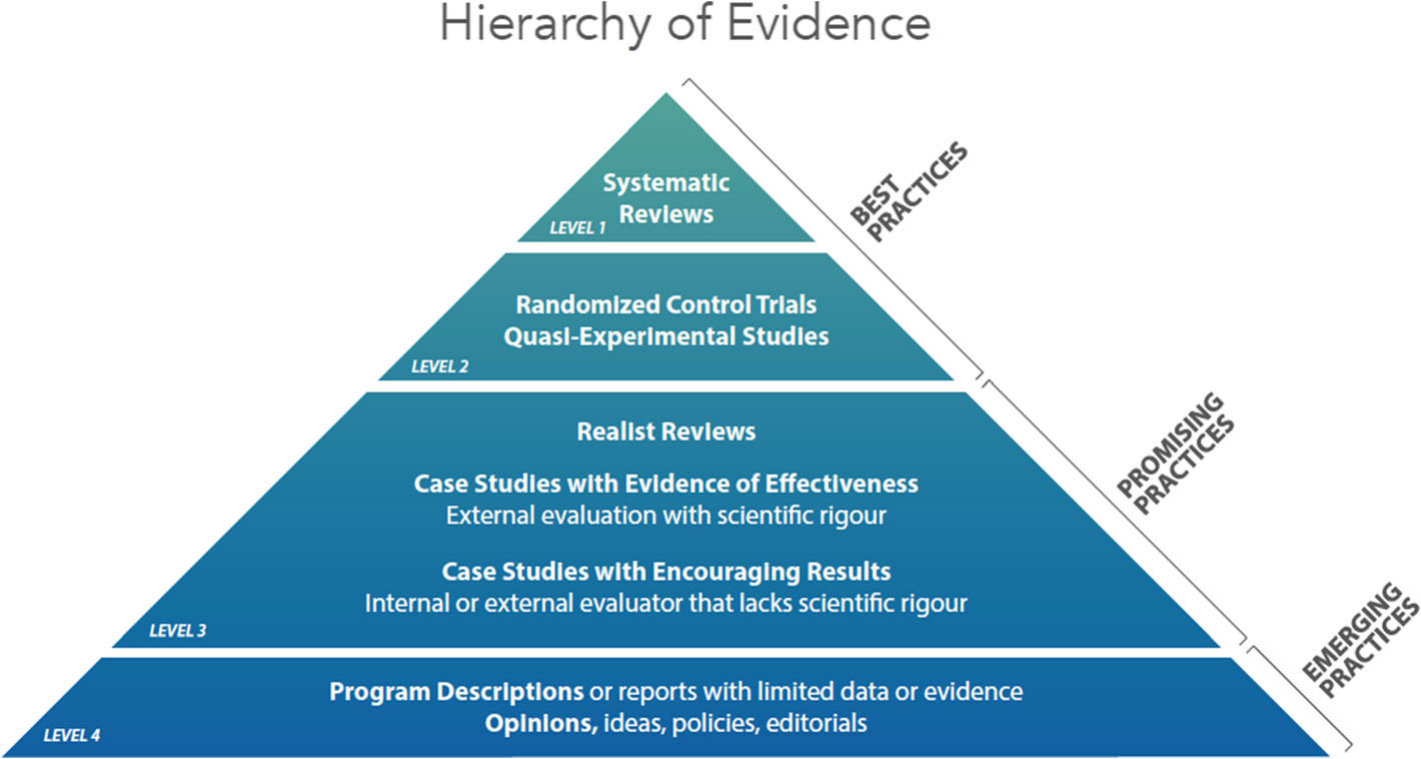
Hierarchy of promising practices evidence

We therefore used the Canadian Homelessness Research Network [31] hierarchy of evidence (Fig. 2) as a transparent mechanism for identifying and categorising study designs to assess the quality of included articles [31]. The hierarchy outlines 3 categories (and 4 levels) of evidence ranging from best practices, promising practices through to emerging practices. A best practice intervention (level 1 and 2) is a method or technique that has consistently been proven effective through a sufficient body of rigorous research. A promising practice (level 3) occurs when there is sufficient evidence to claim that the practice is proven effective at achieving a specific aim or outcome that is consistent with the goals and objectives of the activity or program, and that holds promise for other organisations and entities. Emerging practices (level 4) are interventions that are new, innovative and which hold promise based on some level of evidence of effectiveness or change that is not research-based.

### Data extraction

Thematic analyses were performed to bring together and integrate the findings of multiple qualitative studies [32]. To identify the quantity and nature of available literature, specific information about each of the included studies was extracted. Data included author, year, publication type, country of origin, setting, study design/quality, workforce participants, whether ethical approval was reported, and the study aim, conditions, strategies and impacts. These were tabulated in a summary data extraction table (provided as an Additional file 2: Table S2).

Thematic analysis [32, 33] was also used to elicit the key themes described or evaluated across publications related to the generic enabling conditions, strategies, challenges and impacts of implementing workforce models for Indigenous PHC. Conditions were defined as environments that either facilitated or constrained workforce development and implementation. We applied a social ecological perspective to the definition of conditions, acknowledging that workforce development is influenced and impacted at multiple levels by individual, organisational, community, culture, geographical, economic, institutional and policy factors. Strategies were those initiatives that sought to increase opportunities or prepare and support the workforce development of PHCs. The main overarching themes and related subthemes occurring across the tabulated data were identified, using Braun and Clarke’s [32] six-phase process, entailing: 1) data familiarisation; 2) generating initial codes; 3) searching for themes; 4) reviewing themes; 5) defining and naming themes; and 6) producing the paper. NVIVO Version 10 qualitative software was used to identify initial codes based on the various conditions and strategies that promoted workforce development and implementation. We then identified the multiple levels of conditions based on individual, organisation, community, culture, geographical, economic, institutional and policy factors. Additionally, we searched for strategies that sought to increase opportunities or prepare and support the workforce development of PHCs, and the impacts of the strategies used.

The initial codes provided a starting point from whence further exploration followed. These codes were seen as tentative and were reworked as the analysis continued. In searching for and reviewing themes, the authors identified the four socio-ecological levels which determined the conditions of workforce development, the strategies under three key areas, and four types of impacts. Thus, the identification of various themes enabled the construction of a narrative that emphasises the types of conditions that were necessary for enabling workforce development to occur and strategies that could be used for the development and implementation of PHC. As a result, the findings of the literature review provided under the sections of Conditions, Strategies and Impact, provide an audit trail [34], making key decisions taken throughout the research process transparent, and enabling readers to determine the validity of the findings [34], as the research thread is woven through the narrative.

## Results

**Fig. 3:**
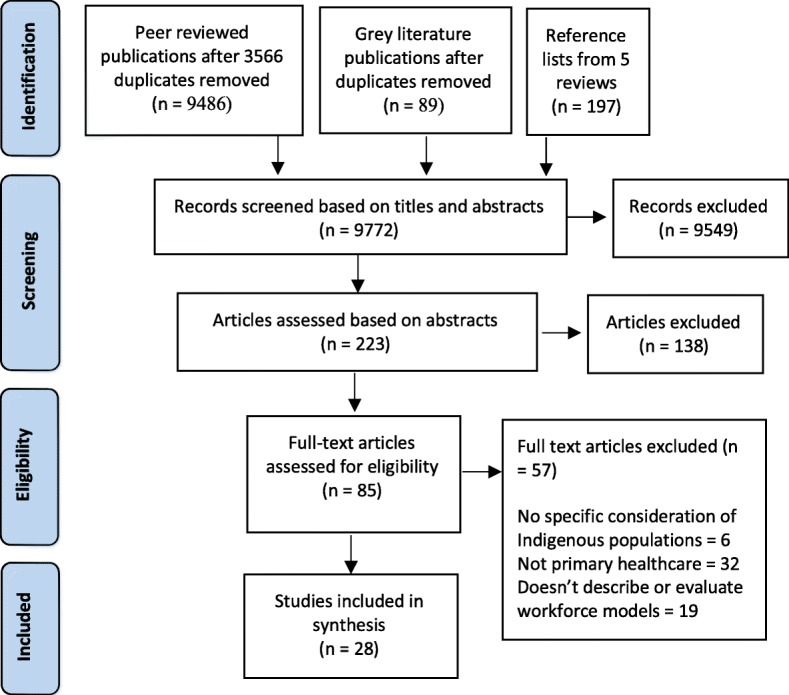
Preferred Reporting Items for Systematic Reviews and Meta-Analyses (PRISMA) statement

The combined searches yielded 9486 peer reviewed publications, 89 grey literature publications and 197 references from five review articles (9772 references). Screening of titles and abstracts resulted in exclusion of *n* = 9549. Two hundred twenty-three publications were considered eligible for further screening to determine whether they met the review’s eligibility criteria. Further rigorous assessment of titles and abstracts led to the removal of 138 publications. Detailed inclusion/exclusion criteria were applied to the full-text assessment of the remaining 85 publications resulting in the exclusion of 57 articles. Twenty-eight publications were included. The results are presented in the Preferred Reporting Items for Systematic Reviews and Meta-Analyses (PRISMA) statement [35] in Fig. 3.

### Characteristics of publications

#### Publication year

**Fig. 4:**
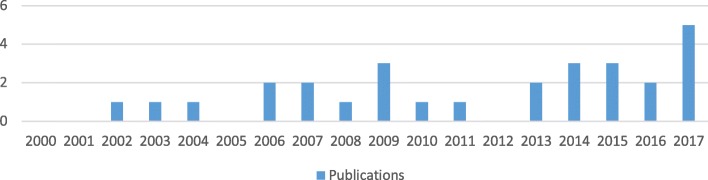
Publications by year

Most included papers were published in the 5 years 2013–2017 (15/28 or 54%) (Fig. 4).

#### Country of origin

The majority of the studies (*n* = 19; 68%) were published by researchers in Australia; five (*n* = 5; 18%) in the USA; and two each in Canada and New Zealand.

#### Study design/quality

No best practice intervention studies (level 1 and 2, Fig. 2) were found. We found 12 studies of promising practices (level 3, Fig. 2) [7, 36–46]. These included: mixed methods studies; cross sectional evaluations; studies based on grounded theory or thematic analyses of interviews, focus groups and/or project or other documents; action research/continuous quality improvement approaches; and a dynamic regression analysis of workforce payroll and financial data across clinics. We also found 16 studies of emerging practices (Level 4, Fig. 2). These were program descriptions, commentaries and concept papers, personal reflections, policy briefs and strategic plans.

#### Workforce participants

**Fig. 5:**
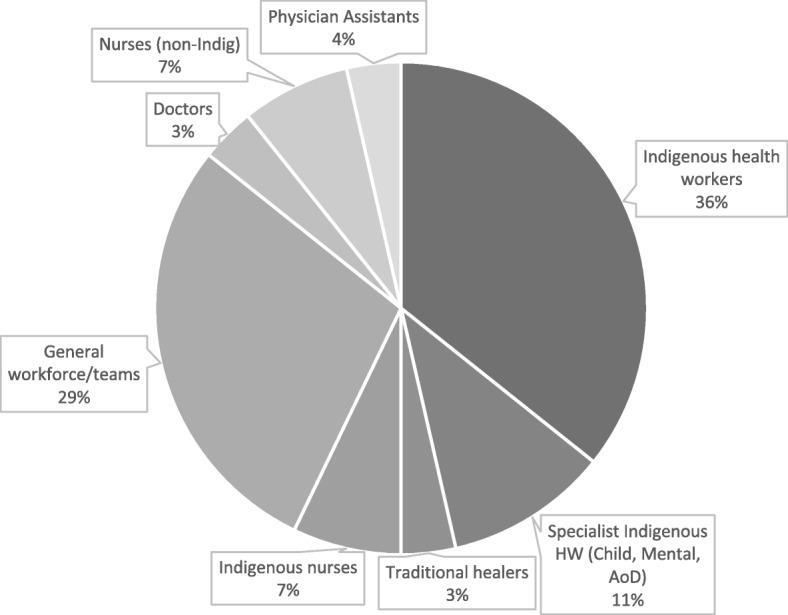
Workforce type

More than half of the studies (16/28 or 57%) focused exclusively on Indigenous health practitioners (Fig. 5). Ten of these studies focused on the roles of Indigenous Health Workers (IHW); three on specialist Indigenous health workers in child, mental health and alcohol and drug work; one on traditional healers; and two on Indigenous nurses. Indigenous health workers were variously named Māori Community Health Workers, Community Health Representatives, Paraprofessional Health Workers, Aboriginal Health Workers and Community Health Workers. The remaining 12 (43%) studies focused on the general (Indigenous and non-Indigenous) workforce or teams within Indigenous PHC (*n* = 8), doctors (*n* = 1), nurses (*n* = 2) and physicians assistants (*n* = 1).

### Characteristics of study interventions

The studies encompassed varied geographical locations (remote, rural and urban) and professional groups, but all 28 studies evaluated or described workforce models that were developed and/or implemented in Indigenous primary healthcare. The studies’ aims, conditions, strategies and impacts summarised in Table 1 below. The X denotes that this element was identified by study authors as an aim, condition, strategy or impact of the described or evaluated study intervention.

**Table 1:** Summary of study aims, conditions, strategies and impacts

	Aim	Conditions				Strategies			Impacts			
	Workforce development/support	Govt. & professional org. Policies	Communities & cultures	PHC policies & environments	Characteristics of health practitioners	Recruitment & retention	Roles, teamwork & capacity	Supervision, mentoring & support	Workforce capacity	Workforce sustainability	Resources to enable growth	Healthcare improvements
(Ahuriri-Driscoll et al., 2015) [47]	X	X	X		X			X				
(Boulton et al., 2009) [48]	X		X	X			X	X				
(Browne et al., 2013) [36]	X			X	X			X	X			
(Chernoff & Cueva, 2017) [37]	X						X				X	
(Conway et al., 2017) [38]	X		X	X	X		X	X		X		X
(Cramer, 2006) [39]	X			X	X		X		X			
(Gampa et al., 2017) [40]	X		X		X		X		X			
(Katz, O’Neal, Strickland, & Doutrich, 2010) [49]	X	X		X	X	X	X					
(Keltner, Kelley, & Smith, 2004) [50]	X	X	X	X			X	X				
(King et al., 2017) [41]	X			X				X	X			
(Laufik, 2014) [51]	X	X		X		X	X					X
(Lloyd et al., 2008) [42]	X	X	X			X	X	X	X			X
(The Lowitja Institute, 2014) [52]	X	X		X								
(Mallee District Aboriginal Services, 2014) [53]	X			X		X		X				
(Minore & Boone, 2002) [54]	X			X	X		X	X				
(Minore, Jacklin, Boone, & Cromarty, 2009) [55]	X	X	X	X			X					
(Murray & Wronski, 2006) [56]	X	X		X		X	X	X				
(Nagel, 2009) [43]	X	X		X			X	X	X	X		
(Nelson et al., 2015) [57]	X		X	X	X		X	X				
(Panzera et al., 2016) [7]	X	X		X		X	X	X	X	X	X	X
(Roach, Atkinson, Waters, & Jefferies, 2007) [58]	X			X		X	X					
(Schmidt et al., 2016) [44]	X	X		X	X		X	X	X			X
(Walker, Tennant, & Short, 2011) [59]	X	X		X			X	X				
(Watson, Young, & Barnes, 2013) [60]	X		X	X	X		X	X				
(Weymouth et al., 2007) [45]	X		X	X	X	X	X	X	X			
(Williams, 2003) [61]	X				X		X					
(Wilson, Magarey, Jones, O’Donnell, & Kelly, 2015) [62]	X					X	X	X				
(Zhao et al., 2017) [46]	X	X		X		X		X		X		

### Conditions

Studies reported the types of conditions that were necessary and sufficient for enabling workforce development to occur. These conditions occurred at four levels: 1) policies of governments and professional organisations; 2) communities/cultures; 3) health service policies and environments; and 4) characteristics of individual health practitioners (see Table 1).

#### Governments’ and professional organisations’ policies

The important role of macro government/professional organisational policies as enablers of workforce development/ implementation was suggested by the frequency of their mention; these conditions were identified in almost half of the studies (13/28 or 46%). Studies identified that it was not only the level and continuity of government funding that facilitated the ability of PHCs to recruit, develop, support and sustain staff, but also its allocation to meeting particular service needs and skills shortages [7, 42–44, 46, 47, 49, 58]. For example, in their evaluation of the role of the health workforce in the implementation of the Northern Territory Preventable Chronic Disease Strategy (PCDS), Lloyd [42] identified that new resourcing facilitated implementation of the policy intent to change and expand Indigenous PHC practice from clinical to population health. However effectiveness of the initiative was limited by a lack of funding dedicated to changing structures services or programs and workforce development to enhance skills congruent with the policy goals [42].

Studies also identified the role of government legislation and/or policy in regulating the recruitment, terms and length of employment, financial accountability requirements, quality of working life, capacity and scope for career development and support for groups of healthcare professionals [47, 51, 52, 56, 59]. Two studies identified the need for improved clarity in government legislation and/or policies concerning the translation of nationally consistent competency standards and qualifications into job specifications and training pathways for IHW [56]; and practice responsibility for nurses and IHW [39, 56]. One study [50] identified the importance of partnerships between national/state/local government departments and PHC to keep Indigenous health issues on the radar.

Three studies identified the importance of professional organisations in establishing and upholding professional standards and advocating for professional groups such as traditional healers and IHW [47, 52, 55]. Professional organisations were able to propose clarity of: job descriptions; standardised training; accredited educational programs; certification of graduates; and regulation of practitioners. For example, Ahuriri-Driscoll et al. [47] evaluated the contracting of traditional healers to provide *rongoā Māori services* by the New Zealand Ministry of Health. The national professional body for traditional healers established standards for practice and professional leadership. Traditional healers tended to practice in an unpaid voluntary capacity but the professional organisation successfully advocated for funding ($1.9 million p.a. across 16 contracts). It was considered likely that formalisation of the *rongoā* through registration and accreditation, would attract additional funding [47]. However, authors noted the incongruence of advocating national standards which may be at odds with the equally important notion of local autonomy [55].

#### Communities and cultures

Community historical, social, political and cultural conditions were also critical enablers of workforce development. Leadership and effective practice by Indigenous nurses were enabled by the brokerage of relationships with local tribal governing bodies in communities and Indigenous health service systems [49, 50]. Clanship or kinship ties and obligations in their home community enhanced trust in the IHW-client encounter [40, 57], although shared histories (with clients) of stressors and social determinants increased Indigenous workers’ levels of stress [57]. Changing local circumstances [55] and changing community priorities necessitated responsiveness in the types of services provided or ways in which workers’ provided them [38, 48, 55]. For example, in the Navajo nation, Community Health Representatives (CHRs) experienced that historical policies as well as personal clanship or kinship affected levels of trust in the patient encounter. CHRs used their knowledge of community and culture to engender trust in the patient encounter as the essential ingredient in providing necessary and quality healthcare services [40]. Such knowledge of community and culture included: information about kinship ties; proper use of the Navajo language; knowledge and encouragement of traditional therapies, religious ceremonies and traditional practices for funerals; and understanding, respect for, and engagement in cultural values and practices. CHRs were also required to cope with grief related to the death of clients on their own and with limited support [40]. Furthermore, the geographical location of the community, particularly remoteness, was an important condition affecting models of workforce management, including distance management, and workforce supply and retention [45, 47, 57].

#### PHC policies and environments

PHC recruitment, support, development and retention policies were identified in 22/28 studies (79%) as conditions that enabled workforce development and implementation. Conditions that enhanced workforce development and/or implementation were: long-term commitment from managers to Indigenous health improvement [42, 43, 53], strong clinical leadership [43], and sound relationships between managers with workers [43]. A lack of management support had detrimental effects including: a lack of enthusiasm for work [45]; ineffective team work [44]; poorly designed electronic patient records or failure to share them [38, 44]; not knowing role expectations [44, 54]; having to prioritise acute care demands over preventive or chronic disease management [42–44, 59]; loss of continuity of care and patient trust [38]; disquiet over the standard of care provided [39, 54]; and staff frustration, stress and turnover [38, 41, 42, 44, 45, 48, 49, 51, 54, 57].

Seven studies highlighted the impact of staff shortages (particularly in remote PHC) on the employment conditions of the remaining workforce. Staff shortages resulted in a heavy reliance on short-term agency-employed nurses and high staff turnover [7, 38, 39, 42, 44, 46, 58]. Partly as a result of workforce shortages, studies described the complexity of roles of remaining workforce groups [42, 43, 48, 49, 51, 58, 60], time pressures in meeting community members’ healthcare needs [38, 44, 47], the need for greater management support [38, 42, 44, 57], the absence of uniformity in training, roles, or conditions of employment [51, 58, 60], and being given leadership roles which staff were not prepared for [49]. For example, a study of nursing practice in a remote Australian community found that managerial, professional and regulatory neglect of the conditions essential for competent nursing required nurses to practice in an amorphous (changing and inconsistent) way. Nurses experienced being ‘dropped’ in the remote area where practice rules are disregarded and ‘no-one sees your practice’; ‘crossing’ or ‘overstepping boundaries’ occurred regularly; and practice ‘outside the scope of nursing’ was expected. Cramer [39] urged nurses to reflect on how they could meet their professional obligations given these workforce conditions, since the consequence was to infringe on the rights of Aboriginal people to adequate standards for safe health care [39].

#### Individual characteristics of healthcare practitioners

Thirteen/28 studies (46%) identified individual characteristics of healthcare workers as enablers of workforce development. Demographic factors, including the Indigeneity of the healthcare practitioner, enhanced their encounters with clients, but also their work/life stress [38, 44]. One study addressed the effect of non-Indigenous health professionals’ attitudes on the quality of healthcare provided [62]. Their motivation to work effectively in Indigenous health was determined by levels of practical knowledge, fear of practicing in Indigenous health, perceptions of difficulty and willingness to learn [62]. For traditional healers, their typically older age was identified as a potential barrier to the sustainability of their workforce [47].

Nine studies described high levels of stress and burnout experienced by individual healthcare workers [38–40, 44, 45, 47, 54, 57, 61]. Stress resulted from other conditions in PHC systems, structures and/or management as well as community/cultural/family responsibilities, but was itself a condition of workforce performance. Its consequences included a reduced staff capacity to invest in strengthening and developing their practice [47], and attrition of IHW and nurses [61]. An example of such workforce stresses was provided by Williams [61] which found that Australian Aboriginal managers had the highest levels of emotional exhaustion, followed by IHW (particularly women). Emotional exhaustion is considered the first stage of burnout and can also be a precursor to physical ill-health. The situation could be exacerbated by pre-existing chronic illness that is highly prevalent in Indigenous communities, including among IHW [61].

Five studies outlined individual characteristics of healthcare practitioners that facilitated effective practice [36, 38, 42, 59, 60]. They included readiness to learn and change practice [36], perseverance and strength in the face of stressful conditions [49], confidence in professional relationships and healthcare knowledge [36, 60], motivation [38, 59] power equality [36], and participation and/or leadership [42]. Katz [49] outlined the explanations of Indigenous nurses for their retention in PHC as commitment to the organisation, ability to resolve problems within the workplace, feeling respected and valued and being able to use independent judgment.

### Strategies

In response to the conditions, three key interrelated and overlapping strategies for workforce development and implementation were identified: 1) enhancing recruitment and retention; 2) strengthening roles, teamwork and capacity; and 3) improving supervision, mentoring and support (Table 1).

#### Enhancing recruitment and retention

Ten studies (32%) incorporated strategies to improve the recruitment of doctors, nurses, IHW and other practitioners to provide healthcare, particularly in remote communities [7, 42, 45, 46, 49, 51, 53, 56, 58, 62]. These included initiatives to improve the pipeline from health practitioner training to practice in Indigenous PHC through mechanisms such as promoting rural health as a career [58], advocacy for funding of salaries [58], and appropriate selection processes in matching registered nurses to communities [45, 53]. A good example of enhancing recruitment strategies was provided by the Mallee District Aboriginal Strategy [53] that created targets for increasing the proportion of Indigenous employees; Indigenous staff representation on selection panels; Indigenous participation in orientation for all employees; Indigenous staff engagement in delivery of face to face workplace orientation; and supporting Indigenous applications for vacancies. As well, the organisation positioned itself as a specialist consulting advisor to other regional organisations and stakeholders [53].

Strategies to retain staff were described in six studies [7, 45, 46, 49, 56, 58]. Retention strategies included extending workforce competencies and skills sets to promote workforce flexibility [56], training pathways to equip IHW for expanded clinical roles and robust career pathways [56]; and supporting advanced training to better equip healthcare practitioners for complex roles in the primary health care system [46, 58]. Other incentives for retention included: management support [49]; attractive leave arrangements, professional feedback, debriefing, professional support and conditions of service [45]; the demonstrated valuing of nurses through use and acknowledgment of their experience in mentoring, policy development, review, decision making and quality improvement efforts; and study assistance and practical incentives [45].

#### Strengthening roles, teamwork and capacity

Twenty-two studies (61%) incorporated strategies to enhance roles including leadership, teamwork and capacity [7, 37–40, 42–45, 48–51, 54–62]. Studies identified issues relating to the definition of professional roles and understanding of practitioners own and others roles [41] within difficult care environments and through team approaches [54]. A Canadian study identified that redefinition of IHW roles was required in response to questions of professional and organisational liability [55]. Studies commented on the need for enhanced role recognition in relation to a variety of professional groups including: IHW [54, 55]; traditional healers [48]; physical assistants [51]; Indigenous nurses [49]; alcohol and drug workers [43]; Indigenous and non-Indigenous child healthcare workers [60]; Indigenous managers and allied health staff [38]; and Indigenous practitioners as cultural brokers [60]. Strategies for ensuring ongoing role development in one study included multi-stage consultation with stakeholders in Canada to determine the scope of IHW practice [48]. Seven competencies for IHW practice were identified: 1) Aboriginal and primary health care; 2) empowerment, community relations and cultural competence; 3) prevention, promotion and protection; 4) emergency care; 5) communication; 6) ethics, leadership and teamwork; and 7) administration. The consultation also proposed that enhanced recognition and clarity of roles be linked to appropriate remuneration [48].

Four studies focused on strengthening leadership by IHW or improving the integration of IHW within interdisciplinary teams to improve the health of their clients. Examples were provided for maternal and child health [37], general healthcare/wellbeing [40] and chronic disease prevention and management [41, 44]. Strategies for reducing the workload of IHW in rural Alaskan communities included shifting the focus of the PHC upstream to patient education about self-care for minor issues. This shift in PHC focus helped to prevent IHW burnout resulting from their frequently being called after hours to provide care to community members [37].

Eight studies stated that teamwork was the only workable means of delivering culturally appropriate health services in remote PHC settings, particularly for chronic disease care [41, 44, 51, 52, 54–56, 59]. Overall, an integrated team-based approach required a shared purpose, creative problem solving, mutual respect for the knowledge base of various professional groups, and acceptance and utilisation of overlaps in respective scopes of practice [42]. A focus on developing identity and cohesion across workforce teams was addressed in relation to the alcohol and drug workforce [43] and more generally in interdisciplinary team work [60]. Strategies that were explicitly linked to role enhancement within teams were the preparation of graduates to function effectively in teams through professional health sciences’ curricula and practice placements [54], access by all team members to electronic patient systems [41, 44], and strategies for efficient use of existing health workforce by the effective deployment or extension of skills [7]. However, as Minore [55] noted, interdisciplinary healthcare teams often failed to build a common spirit and morale among members. For example, In remote Australian communities, Schmidt et al. [44] found that the confidence and capacity of IHW to provide chronic disease care and service coordination was enhanced by ongoing support by an Indigenous Clinical Support Team, communication of the IHW role to team workers, training to support the IHW role and IHW knowledge of their clients and environment. However, team work would have been improved by a greater emphasis on engaging clinical leaders and local champions about the IHW role in chronic disease care [44, 57].

Professional development to lift the educational and formal health care training levels of existing employees and/or expand opportunities for new workers was identified in seven studies [38, 43, 45, 47, 50, 52, 59]. Diverse training pathways were described, with studies finding that no single pathway was likely to meet all practitioners’ needs (e.g. [47]). For some, formal institution-based curricula and certification pathways were considered appropriate (e.g. [47]). Indigenous practitioners (such as traditional healers) preferred a dual system incorporating both cultural guidance and support as well as institution-based learning, or an apprentice-style learning system that was consistent with ‘traditional’ oral knowledge transmission to emphasise an Indigenous worldview and cultural knowledge [47].

For non-Indigenous practitioners, the need for cultural education to minimise discrimination and distrust and work towards providing and maintaining culturally safe environments was highlighted as important to preventing cultural mishaps, caused through unintentionally disrespectful practice (e.g. [38]). Management strategies for enhancing workforce capacity included providing training opportunities that were relevant for career advancement, supervision [42–44, 48, 59], and implementing dedicated chronic disease positions [42]. Formal skills acquisition [47], registration with a professional body and/or accreditation [47, 50] were also recognised as means of professional advancement and enhanced remuneration.

#### Improving supervision, mentoring and support

Strategies to improve supervision, mentoring and/or support to health practitioners were identified in twenty (71%) studies (Table 1). Two studies outlined the value of clinical supervision. Nagel [43] described regular clinical supervision and clinical review provided to Australian alcohol and drug workers. A model of centralised executive support and peer support were both effective for the remote workforce [43]. Similarly, Nelson [57] identified four different models for effective supervision of Indigenous Australian mental health workers. These included: 1) cultural supervisors (an Indigenous person with extensive cultural knowledge and capacity); 2) dual supervisors (one with demonstrated proficiencies in professional development and one that balanced professional and community/cultural obligations in service provision); 3) consultation (where a clinical skills expert provided didactic and skills-based training and sometimes provided additional case consultation/clinical supervision); and 4) communities of practice through modern technologies (particularly for remote-working practitioners). The three essential components for effective supervision were: clinical expertise, personal support recognising the specific issues faced by Indigenous practitioners, and cultural/community understanding [57]. The authors concluded that investment in best-practice supervision could reduce the costs of cyclical workforce recruitment and unmanaged mental illness of clients due to workforce gaps [57].

The only included study of a formal workforce development mentoring strategy established a framework based on reciprocity and equality between Australian IHW and non-Indigenous allied health professionals [36]. Mentoring partnerships worked most effectively when both parties were comfortable in their roles as both teacher and learner. Power differences between mentoring partners were detrimental to the relationship. Another study identified the potential for tele-mentoring strategies using existing satellite facilities in remote Indigenous communities [59]. Formal workforce support was described in one study. For example, in the Navajo nation, the integration of CHRs into clinic-based care teams was supported by The Community Outreach and Patient Empowerment (COPE) Program that established improved referral processes, case management meetings, and supported joint home visits and CHR access to electronic health records. Patients were enrolled either by the CHR or via provider referral; CHRs had flexibility in who they chose to enrol, based on their perceptions of who might benefit. In particular, the ability of CHRs to access the Electronic Health Record to document their encounters and obtain clinical information on their clients was an important factor for establishing stronger clinic-community linkages. Nonetheless, the CHR experience of these programmatic efforts suggested that further work was needed, particularly to integrate care teams across the continuum of clinic- and community-based providers [41].

Informal workforce development support was outlined in three studies. Conway [38] described IHW support structures such as group meetings and debriefing sessions. Implementation champions were identified as “go to” persons and activities were developed to enhance IHW empowerment and knowledge sharing. Weymouth [45] found that the management support given to remote nurses after a critical incident was poor, but that the Bush Crisis Line provided professional support and was highly regarded. Wilson et al. [62] presented a model for exploring non-Indigenous health professionals’ attitudes to practice in Indigenous PHC. It was proposed as a useful basis for self-reflection on levels of confidence, attitudes, characteristics, experiences, approaches and assumptions about Indigenous health, as an important precursor to future practice. The model was proposed as a framework to facilitate group discussions between all health professionals about working together in Indigenous health [62].

### Impacts

Because many of the studies were program descriptions and/or commentaries, only 12/28 studies (43%) identified impact from workforce related interventions. Four types of impacts were identified: 1) workforce sustainability; 2) workforce capacity; 3) resources/growth; and 4) healthcare improvements. No studies identified any impacts relevant to policy initiatives or measures.

#### Workforce sustainability

Four studies identified impacts related to workforce sustainability. Two studies reported sustained retention of staff and a stable workforce [7, 43]. Nagel [43] found that 20 new positions established to comprise a new Remote Alcohol and Drug Workforce in Australia had been filled after 3 months by Indigenous workers with Certificate level qualifications, and of those recruited, almost all stayed. This was attributed to support provided to workers in both personal and practical ways such as: professional development, peer support, advocacy as a group, career structure, and travel and accommodation support. Panzera [7] reported improved effectiveness in relation to workforce recruitment and retention. They reported that a stable and sustainable local workforce was developed through strengthening health systems and workforce training solutions e.g. task substitution and redistribution.

The other two studies reported an absence of sustainability in their staff retention [45, 46]. Weymouth [45] found that registered nurses working in remote PHC that were supported through distance management were dissatisfied with infrastructure, support and management, but satisfied with their roles. Dissatisfaction with management support increased staff frustration and stress and prompted staff turnover. Also from Australia, Zhao et al. [46] found that despite substantial increases in resourcing in remote PHCs, health service models were not sufficiently robust to sustain the supply and retention of resident health staff. In this case, PHCs resorted to a heavy reliance on short-term agency employed nurses and high turnover of government employed staff [32].

#### Workforce capacity

Four studies (37%) identified an impact that was broadly related to the capacity of the workforce [7, 36, 40, 41]. Three/4 of these studies found enhanced IHW leadership capacity [36, 40, 41]. Browne [36] found that an Australian mentoring workforce development strategy for IHW and non-Indigenous allied health professionals demonstrated capacity to achieve an increased skill base of IHW; cultural safety among non-Indigenous health professionals; and effective infrastructure, leadership and partnerships. Two-way learning and development occurred; IHW and non-Indigenous allied health professionals reported that they met their identified learning needs [36]. One Navajo study found that enhancement of the culturally specific factors that build and sustain the CHR-client interaction resulted in improvements in communication, respect for clients and client empowerment [40]. Another Navajo study found that a chronic disease healthcare workforce empowerment and support program resulted in CHR perceptions of strengthened validity and reputation, enhanced ability to positively affect health outcomes, and improved ability to deliver health coaching to clients. Eighty percent (80%) felt strongly positive that monthly work-based training sessions in CHR-provider relationships, motivational interviewing, self-care and wellness, and team-building were useful and 45% felt communication and teamwork had improved [41]. The other study found enhanced capacity of the general workforce [7]. Panzera [7] found that workplace planning based to address specific workforce skills shortages led to the delivery of locally-relevant workforce training solutions, and extended competencies and skills sets to facilitate task substitution and redistribution.

#### Resources to enable growth

Impacts in resourcing and growth were identified in two studies (7%) [7, 37]. Panzera [7] found that participatory regional health workforce planning processes in regional Australia accurately modelled current and projected local workforce requirements, and led to an increase in delegated practice models. Chernoff [37] found that the maternal and child healthcare model delivered to Alaskan Native people living in rural communities was translatable to other tribal and limited-resource contexts. In part, transferability was attributed to its delivery by IHW; but also because the model was tailorable to local context and suited for regions with limited infrastructure and otherwise underserved families and individuals [46].

#### Healthcare improvements

Finally, six studies reported the effects of workforce strategies on healthcare outcomes [38–42, 44]. Conway 38] found that IHW implementing the Flinders Closing the Gap chronic disease self-management support program could have been better supported and supplemented, but the IHW reported that the program itself was appropriate, flexible and acceptable [38]. King [41] found that an empowerment and support program in the Navajo nation enhanced the ability of CHR teams to improve clinic-community linkages for chronic disease prevention and management [41]. This occurred primarily through strengthened collaborations between Public Health Nurses and CHRs, and access to electronic health records. Gampa [40] found that communication was improved in the IHW-client interaction when IHW utilised culturally-specific knowledge and practices, and clients became more empowered.

The other three studies found that in the absence of a supportive service model, nurses and IHW were unable to facilitate improved healthcare. From remote Australian communities, Cramer [39] claimed that managerial, professional and regulatory neglect of the conditions essential for competent nursing meant that Aboriginal people did not receive the basic standards for safe health care. Schmidt et al. [44] reported that IHW were most able to strengthen systems and practice where they had skills and knowledge i.e. client self-management support and linking with community and other services and resources. They found that a skilled, dedicated and satisfied IHW workforce was accompanied by client satisfaction. But despite their competence, capacity, and client satisfaction, they were unable to address all of the systems’ issues that were barriers to best practice chronic care. Also working in remote Australian communities, Lloyd [42] found that the IHW workforce tended to implement aspects of chronic disease policy that drew on their existing skills and avoided or delayed implementation that required new skills. Because workforce issues were not addressed, policy recommendations were only partly implemented.

### Discussion

The review found only 12/28 (43%) studies that provided any evaluation of the workforce strategies and most of these studies used weak study designs; there is consequently little definitive evidence of the effects of particular strategies to guide practice. We found no best practice intervention studies, 12 studies of promising practices, and 16 of emerging practices. Our overall impression of the literature was that commentaries and policy documents that described the domains of best practice workforce development and implementation were plentiful. But there was a dearth of studies that examined *how* best practice should be achieved, or what worked to improve workforce sustainability, capacity, resources to enable growth or healthcare improvements. There was also a significant heterogeneity in the strategies and outcomes, making comparisons of intervention effects difficult. We cannot therefore make categorical recommendations about particular strategies for PHC workforce development and implementation.

There is the possibility that the review did not locate all relevant studies, although a rigorous and thorough search strategy suggests that this was not the case. Many existing publications may not be available in key international databases [63]. The authors of the review are based in Australia with extensive knowledge and experience in Indigenous health research in the Australian context. Because of this direct knowledge and experience, several known databases specific to Australian Indigenous health research were searched. Similar Indigenous specific databases from other included countries are unknown to the reviewers. This may have resulted in a bias towards Australian studies. It is also possible that relevant intervention descriptions or evaluations may have been misclassified; however, the high level of agreement between blinded coders, and consensus on all included studies also suggests not. Evaluations with positive findings are more likely to be published. Therefore it is possible that the published evaluations reviewed overestimate the true effectiveness of PHC workforce development interventions for Indigenous peoples [64].

**Fig. 6:**
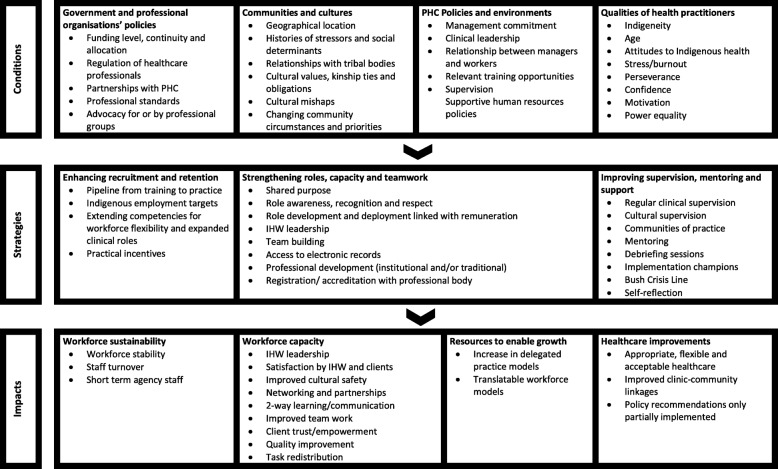
Framework for Indigenous PHC workforce development and support

Despite these limitations, the reviewed studies can be used to inform workforce development decisions. They suggest that an optimal sustained, capable and growing workforce model requires strategies for enhanced recruitment and retention; strengthened roles, capacity and teamwork; and improved supervision, mentoring and support. In turn, these strategies are enabled by government funding and appropriate regulation, support and advocacy by professional organisations; community engagement; PHC leadership, supervision and support; and practitioner Indigeneity, motivation, power equality and wellbeing. These findings have been used to develop a framework for Indigenous PHC workforce development and support (see Fig. 6).

Studies that reported outcomes of sustained staff retention and a stable workforce reported the important enabling conditions as management commitment, workforce training and strengthened health systems [7, 43]. However, only 2/12 studies reported sustained staff retention as an outcome [7, 43]. Processes such as participatory continuous quality improvement were useful for navigating such changes (e.g. 7). Such interactions between personal, professional, organisational and contextual factors in efforts to improve service delivery have been noted in other reviews of Indigenous PHC workforce development [4, 22, 23], and in workforce change efforts internationally [8].

The conditions important for improving workforce capacity were enhancement of cultural values, kinship ties and obligations; management commitment and leadership; relationships between managers and workers; training opportunities; supervision; supportive human resources policies; and power equality. Three of the four studies that found improved workforce capacity [7, 36, 40, 41] focused primarily on the capacity for team leadership by IHW. The importance of enhancing the capacity of Indigenous staff, including IHW, is suggested by international studies that found that people prefer to visit health professionals from the same ethnic background [17, 65]. The critical clinical functions of IHW in CANZUS nations include: first point of contact; liaison and cultural brokers; promoting health; community and/or clinical care; administration; policy development and program planning. Indigenous health professionals can align their unique technical and sociocultural skills to improve patient care, improve access to services and ensure culturally appropriate care [66, 67]. Yet studies documented a lack of understanding or recognition of their potential leadership roles within teams, high levels of stress, and typically low payment. Like other literature reviews [4, 25], studies described strategies for strengthening IHW team leadership roles in preventive health education; ensuring their access to electronic client records and inclusion in case management collaborations within chronic care teams; mentoring and supervision; and pathways to training and qualifications, including for task substitution and redistribution. Two-way mentoring between IHW and allied health practitioners was a notable strategy. Mentoring was fund to enhance two-way empowerment and potentially of healthcare performance. These findings are consistent with that of a recent review of mentoring initiatives to enhance Indigenous health, education, employment and justice system capacity [68]. They are also consistent with international evidence that staff development needs to be closely linked to service needs [8].

The conditions that supported resourcing and growth were planning for funding levels and continuity, and flexibility caused by delivery by IHWs. The two studies that identified enhanced resourcing and growth [7, 37] suggested that it was not only the resourcing of PHC systems enhancements that were important to developing improved chronic disease care, but also the allocation of funding to remedying particular skills and capacity shortages. As for the Indigenous child protection sector, empowering participatory planning processes were effective in PHC for accurately modelling current and projected local workforce requirements and skillset requirements [69, 70]. For PHC, participatory planning led to an increase in delegated practice models [7].

Finally, the conditions that led to enhanced healthcare performance were effective management and clinical leadership, access to systems such as electronic health records, the relationship between managers and workers and between workers and clients, and the utilisation of culturally-specific knowledge and practices. Three studies found that workforce strategies were effective in enhancing chronic disease and other healthcare performance [38, 40, 41]. Findings of included studies suggested, for example, that chronic disease management will not be optimised unless workforce issues are addressed [42], but that it is also necessary to simultaneously address systems issues [44]. As found in other Indigenous community studies (e.g. [71, 72]), workforce support facilitated the successful strengthening of systems and practice where IHW had skills and knowledge, but team support was unable to address all barriers to systems improvement. Such efforts in improving the Indigenous welfare workforce have also found that a long-term commitment and ongoing support are required to enhance the empowerment of workers and clients [71, 73]. For example, MacFarlane et al. [8] predicted that the success of strategic human resource management in the UK public healthcare sector would, in part, be due to the extent to which national policymakers were willing to implement a responsive systems-based model of health service change, with attention to the inter-relationships between the different parts.

## Conclusion

The dearth of evidence about Indigenous workforce models makes it challenging to determine what models and systems support the effectiveness, sustainability and/or growth of the Indigenous PHC workforce. There was little definitive evidence of the effects of particular strategies to guide practice. The findings of this review suggest that the important and complex work of the PHC workforce in improving Indigenous healthcare and health outcomes can be enabled by the policies of governments and professional organisations, community and cultural factors, primary healthcare organisations’ policies and environments and individual qualities of health practitioners. Strategies include enhancing recruitment and retention; strengthening roles, capacity and teamwork; and improving supervision, mentoring and support. But this review suggests that it is not easy to facilitate an optimal sustained, capable and growing workforce model that can confidently improve Indigenous PHC performance. Improvement is needed in the quality of evidence relating to workforce retention/sustainability and growth, and the contribution of the workforce to enhancing healthcare and health outcomes.

## Supplementary information

**Additional file 1.** Table of study characteristics. A table including: author; year; publication type; country; setting; study type and quality; participants; aim; conditions; strategies; and, impacts.

**Additional file 2.** Medline search 1. An example search to demonstrate the search strategy.

## Data Availability

All data generated or analysed during this study are included in this published article [and its supplementary information files].

## Abbreviations

CANZUS nations: Canada, Australia, New Zealand and the USA
IHW: Indigenous Health Workers
PHC: primary healthcare service
PRISMA: Preferred Reporting Items for Systematic Reviews and Meta-Analyses
