# Supplementary material for: Working well: a systematic scoping review of the Indigenous primary healthcare workforce development literature
Source: BMC Health Serv Res. 2019 Oct 29;19:767. doi: 10.1186/s12913-019-4580-5 (PMC6819619; doi:10.1186/s12913-019-4580-5)
Supplement: Supplementary file 1 — Additional file 1. Table of study characteristics. A table including: author; year; publication type; country; setting; study type and quality; participants; aim; conditions; strategies; and, impacts. [file 12913_2019_4580_MOESM1_ESM.docx]

**Additional file 1: Table of study characteristics**

| **Author**  **Year**  **Publication type** | **Country Setting** | **Study type and quality** | **Ethical approval?** | **Participants –and focus on Indigeneity?** | **AIM** | **Conditions of the Intervention** | | | | **Strategies** | | **Impacts** |
| --- | --- | --- | --- | --- | --- | --- | --- | --- | --- | --- | --- | --- |
|  |  |  |  |  |  | **Government and prof org policies** | **Communities/ cultures** | **Health service policies & environments** | **Qualities of health practitioners** |  | |  |
| Ahuriri-Driscoll (2015)  Peer reviewed paper | NZ | Point in time survey  Emerging practice (Level 3) | Not reported | Traditional healers  Yes | To report traditional healers’ aspirations for professional development and training | N.Z. government strategies reflected dual training priorities. Importance of advocating for professional groups such as traditional healers. | Identified the influence of geographical location. Other family/community roles and responsibilities. | Most are volunteers who work part-time or casually. Financial constraints impact capacity to invest in strengthening and developing practice. | The traditional healing workforce is older than the general workforce; leaving means taking their knowledge and experience with them. | No single training pathway will meet all practitioners’ needs. Apprentice-style learning consistent with ‘traditional’ oral knowledge transmission is preferred; knowledge of te reo, mātauranga and tikanga of highest priority emphasising a Māori worldview and **deep** cultural knowledge. Healers advocated for a dual system - cultural guidance and support and institution-based curricula and certification. Formalisation of rongoā through registration, accreditation, will promote additional funding. | |  |
| \| Boulton  (2009)  Peer reviewed paper \| \| --- \| | NZ | Qualitative data from 2 projects and concept validation through co-authorship by a current practicing community health worker  Emergingpractice (level 3) | Not reported | Māori community health workers  Yes | To outline the role Māori community health workers (CHWs) play in the delivery of whānau ora services and explore the tensions and challenges faced by these workers in carrying out their role |  | Ambiguities resulting from differing expectations between contractual requirements of PHC, First Nation communities’ priorities, and CHR's own expectations result in stress for CHRs. | CHRs training opportunities are short-term, ad hoc, specific to a health provider's needs and offer limited career advancement options. Roles complex, varied and in high need communities. |  | Greater recognition of the unique role they play in the delivery of public health and PHCs, linking this recognition to appropriate remuneration and ensuring ongoing role development is met within a Māori worldview. | |  |
| Browne (2013)  Peer reviewed paper | Aust | Post-program evaluation – qualitative  Promising practice (Level 3) | Yes | IHW and allied health  Yes | To evaluate a mentoring workforce development strategy for Aboriginal Health Workers and non Aboriginal allied health professionals. |  |  | Capacity to improve practice facilitated through organisation and management support. | Mentoring most effective when comfortable as teacher and learner. Power differences detrimental. Readiness to learn, personal attributes => improved practice. | Framework is established based on equality and true reciprocity | | Demonstrated capacity for: 1) increased skill base of IHWs; 2) cultural safety of non-Aboriginal health professionals; and 3) effective infrastructure, leadership and partnerships. Two-way learning and development. IHWs and non-Aboriginal health professionals reported meeting their identified learning needs. |
| Chernoff (2017)  Peer reviewed paper | USA  (Alaska) | Qualitative grounded theory study  Promising practice (Level 3) | Yes | Tribal HW  Yes | To explore the interactions that Community Health Aides/Practitioners (CHA/Ps) have with mothers, infants, families, and teens |  |  |  |  | CHA/Ps unique role as on-the-ground health and wellness resource in their communities, they consult with other medical professionals to provide care in rural Alaska. CHA/Ps provided prenatal care, patient education, emergency delivery services, well-child visits, and outreach to teens. Patient education as a primary responsibility reduced patient load and prevented burn-out. | | Healthcare delivery model is translatable to other tribal and limited-resource contexts. |
| Conway (2017)  Peer reviewed paper | Aust | Qualitative thematic analysis of interviews  Promising practice (Level 3) | Yes | IHW  Yes | To explore IHWs’ perceptions of the effectiveness and appropriateness of the Flinders Closing the Gap  program, and the barriers and facilitators that IHWs experience in their workplace and communities in providing self-management support. |  | Differing expectations between contractual requirements of PHC, First Nation communities’ priorities, and the CHR's own expectations result in stress for CHRs | Staff turnover => loss of continuity of care and patient trust. Competing priorities impact CHR performance. Stress and burnout from time pressures, lack of support, poor information systems, and high staff turnover. Contributed to attrition. | Thirst for mentorship and camaraderie among IHWs, and with non-Indigenous workers who required cultural education to prevent cultural mishaps.  Poor life and work balance => workers on the job round-the-clock. | IHW support structures e.g. group meeting and debriefing sessions,  Implementation champions “go to” person to support IHWs. Client and worker empowerment, and activities around sharing knowledge.  Adequate Indigenous representation in management and allied health positions. Cultural education to non-IHWs to minimise discrimination and distrust, and work towards  providing and maintaining culturally safe environments | | IHWs reported that the Flinders Closing the Gap  Program was appropriate, flexible and acceptable.  Recommendations focus on supporting and supplementing the role of IHWs. |
| Cramer (2006)  Paper in peer reviewed journal | Aust | Qualitative participant observation, interviews, documents, and daily chronological recording of field notes, analysed thematically  Promising practice (Level 3) | Yes | Nursing  No | To describe how nursing is practised in a remote Aboriginal community |  |  | Managerial, professional and regulatory neglect of the conditions essential for competent nursing led to an amorphous practice (changing and inconsistent nursing practice). | Nurses' disquiet over the standard of care they provide under adverse conditions.' Nurses attempt to cope with impossible demands and no systems to support a safe and effective health service. | Amorphous practice from 1) detachment (being 'dropped' in the remote area where 'suddenly' practice rules are disregarded and 'no-one sees your practice'), 2) diffusion (the loosely outspread boundaries of the nurses' role), and 3) beyond the nursing domain (practice outside the nurses' formal authority; 'crossing' or 'overstepping boundaries' and 'outside the scope of nursing'). Need introspection on professional obligations where even basic clinical services are lacking. | This study exposes infringements on the rights of Aboriginal people to basic standards for safe health care. The arbitrary expansion of the nurses' role into a medical domain increases nurses' vulnerability to risk, and, in turn, nursing is denigrated. | |
| Gampa (2017)  Peer-reviewed paper | USA | Qualitative grounded theory based on participant observation, interviews, documents, and daily chronological recording of field notes  Promising practice, (level 3) | Yes | Community Health Representatives  Yes | To investigate the culturally specific factors that build and sustain the Community health representative (CHR)-client interaction |  | Historical policies affect trust within the Navajo healthcare encounter. Fundamentally linked to understanding, respect, and cultural values, and particularly having clanship or kinship ties. |  | CHRs need to respect diverse traditional and social practices and use their knowledge of community and culture e.g. language, religious and traditional beliefs. Must also cope with grief with limited support. | CHRs are community members that provide healthcare services to their communities. Trust is essential and enhanced by sharing information about kinship; encouraging traditional therapies or religious ceremonies; appropriate use of the Navajo language;  connecting with families at funerals and engaging in healing. | Improvement in  communication and health, as well as client empowerment and respect for clients. | |
| Katz (2010)  Peer reviewed paper | USA | Qualitative interpretive phenomenological methodology based on nurses telling about  their experience in interviews  Emergingpractice (level 3) | Yes | Indigenous Nurses  Yes | To describe the experiences of Native American nurses working in their tribal communities to address retention | Chronic underfunding of the Indian Health Service related to lack of Native American nurses | Indigenous nurses who return to their communities need mentoring and support to build positive, cooperative relationships and to negotiate with tribal governing body, tribal health administration, and colleagues from other tribal programs. | Indigenous nurses not prepared for leadership roles. Challenges of introducing new technology and ideas into traditional culture. Frequent organisational and policy changes, poor communication about changes, and cut off from information and decision-making power if verbalise concerns. Often led to quitting, an illness, or both. | Strong desire to help their people through prevention and education. Concerns about being respected, able to practice with autonomy. Themes 1) paying the price to fulfil my mission, 2) being and connecting holistically, and 3) transcending the system. Perseverance and strength, despite stressful conditions. Loss if inability to stay or work effectively. | Good system required communication, leaders, and respect. Being effective required autonomy, creativity, and helping their people, especially in prevention of illness, in a holistic way unique to being a Native American nurse. Reasons for staying in a position were commitment to the organization, ability to resolve problems, feeling respected and valued and being able to use independent judgment. Cultural expectation that nurses “be available day and night” needs balance with nurses caring for themselves and families. Administrators need to support new nurses to adapt to working in transcultural setting; turnover tracked with exit interviews. Tribes might provide orientation to communities and mentoring in leadership. |  | |
| Keltner (2004)  Commentary paper in peer reviewed journal | USA | Commentary paper  Emerging practice (level 4) | Not reported | Nursing  No | Leadership | Relationships developed with US county and state health partners to keep health disparities and American Indian health issues on the radar. | Indigenous nurses who return to their communities need mentoring and support to build positive, relationships with the tribal governing body, tribal health administration, and colleagues, and to negotiate tribal and Indian Health Service systems. |  |  | Nursing leadership on the Reservation - public health interventions specific for American Indian populations. Nursing staff supported in professional growth through continuing education, research, and professional membership opportunities. Active participation with local, regional, and state committees and teams. |  | |
| King (2017)  Peer-reviewed paper | USA, Navajo | Mixed methods, cross sectional evaluation of a longitudinal cohort study  Promising practice (level 3) | Yes | Community health representative  Yes | To explore how the Community Outreach and Patient Empowerment (COPE) Program has effected Community Health Representative (CHR) teams over the past 6 years and their ability to improve chronic disease prevention and management |  |  | Limited communication between CHRs and physicians. CHRs had no access to Electronic Health Records for patients; in part due to logistic, training, data accessibility and data sharing issues. High provider turnover  contributed to lack of awareness of CHRs among  providers at some sites. |  | COPE Program works to better integrate CHRs into the local health care system through training, strengthening care coordination and standardized culturally appropriate health promotion materials for CHRs to deliver to clients. Patients are enrolled either by the CHR or via provider referral; CHRs have flexibility in who they choose to enrol. COPE involves providers in training and curriculum development, establishes referral processes, case management meetings that involve CHRs and hospital providers, supported joint home visits, enabled CHR access to electronic health records to document encounters and obtain clinical information on their clients. Nonetheless, further work is needed to  integrate care teams across the continuum of clinic- and community-based providers. | Improved clinic-community  linkages, primarily through strengthened collaborations between Public Health Nurses and CHRs, and access to the  Electronic Health Records. CHRs perceive a strengthened validity and  reputation, enhanced ability to positively effect health outcome, improved ability to deliver health coaching to clients. 80% feel strongly positive that COPE trainings are useful; 45% felt communication and  teamwork had improved. | |
| Laufik (2014)  Personal perspective “special article” in peer-reviewed journal | Aust | Personal reflection/ case study of a PA working with Aboriginal people in Australia  Emerging practice (level 4) | Not reported | Physician Assistants  No | To describe the experience of a Physician assistant (PA) employed in rural Aboriginal Health Services in North Queensland | Government legislation and policy define availability, capacity of and support for healthcare professionals. |  | Pilot evaluations found PAs successful in improving quality of work life for doctors. Team approach is effective in chronic disease management. IHWs have no uniform training, roles, or employment conditions, but are vital for ﬁrst point of contact, liaison and cultural brokerage, health promotion, community care, administration, policy development, program planning. |  | Extending doctor role by task delegation to the PA has potential to improve access for more patients to higher quality care. Advantages: reducing doctor fatigue and isolation, increasing income into practices; potential career pathway for IHWs. Learning the Medicare systems for appropriate care delivery and the systems of an individual clinic was manageable and doctors had more time for other medical tasks.  Downside for PA was frustration of being constrained from fully using skills, including ordering tests, making specialist referrals, and prescribing, that all require attention of supervising doctor. |  | |
| Lowitja Institute, (2014)  Policy brief | Aust | Policy brief  Emerging practice (level 4) | Not reported | IHW  Yes | To describe the drivers of Indigenous health career development and propose a framework | Effectiveness of Australian government’s policy intent to expand Indigenous PHC practice from clinical to population health limited by lack of funding allocation to workforce skills congruent to the policy goals. | Various historical, social and economic community conditions affected the workforce. | Occupational stratification within teams. Workload (patient load, performance indicators and targets, working hours) and workload management (e.g. staff: patient ratios, rostering and work allocation). |  | Behaviour of employers, and workplace cultures they create and foster, shape desirability of place of employment for health workers. Skill development, provision for career development. Human resource management practice, workload management tools, and recruitment and retention policies for sourcing, recruiting and managing labour, employment stability, and composite of skills. |  | |
| Lloyd (2008)  Peer-reviewed paper | Aust | Qualitative thematic analysis  Promising practice Level 3 | Yes | General workforce  No | To explore the role of the health workforce in the implementation of Aboriginal health policy | Australian government policies influence worker recruitment to health, terms and length of employment, financial accountability, quality of working life, and scope for career development. Professional organisations establish and uphold standards. |  | Implementation enabled by additional staff, training, managers commitment, and dedicated chronic disease positions. Barriers were insufficient providers, little support for IHWs, high turnover. Need more Indigenous practitioners to support IHWs and decrease turnover. | Members participate and/or take leadership responsibilities because of expertise as individuals or relationship with a client, not simply because of the position they hold. | Good teamwork: shared purpose, creative problem solving, mutual respect for each other’s knowledge base, as well as acceptance and utilization of overlaps in respective scopes of practice. Education in the general principles of interdisciplinary practice is insufficient preparation for practice in rural or remote places. Policy needs to include strategies for strengthening the workforce; significant changes in staff composition and roles, and organisational support. | Policy was only partly implemented because workforce issues were not addressed. Workforce tended to implement aspects of the policy that drew on existing skills and to avoid or delay implementation that required new skills. | |
| Mallee District Aboriginal Services (2014)  Employment strategy report | Aust | Strategic plan  Emerging practice (level 4) | Not reported | General Workforce  No | To describe Mallee District Aboriginal Services commitment to Closing the Gap by demonstrating its Workforce strategy |  |  | MDAS as a strong employer of Indigenous people and specialist consulting advisor to other organisations. |  | Created targets for % employees identify as Indigenous; participation across all levels; representation on every selection panel; participation in orientation for all employees; engagement in delivery of face to face orientation; increased applications for vacancies. Increase quality student placements. Break down barriers for workforce entrants. Demonstrate organisational commitment to integrating Aboriginal culture and values. Establish interdepartmental and cross organisational mentors and peer support/ mentoring for outreach workers. Establish a study culture and facility. Develop and facilitate cultural awareness training. Provide consultancy services to increase Aboriginal Employment regionally. |  | |
| Minore (2002)  Peer-reviewed paper | Canada | Commentary  Emerging practice (level 4) | Not reported | Professional/ paraprofessional health care teams  Partly | To identify factors fundamental to effective interdisciplinary team functioning including potentiality and challenges. |  |  | Interdisciplinary healthcare teams often fail to build morale. Barriers linked to understanding own and other’s roles while working in a difficult care environment. Professional programs don’t prepare graduates to function effectively in teams. | Not knowing role expectations is the major source of frustration among IHWs. Internal conflict is felt by individuals who are not doing what they think they should be doing. | Need further instruction to ensure that the full potential of the health human resources available— professional and paraprofessional—are realized and applied to meet the needs of otherwise underserved client populations. The information on interdisciplinary practice in all health sciences’ curricula should be extended to include the roles and responsibilities of paraprofessionals—to focus on the whole team. This learning should occur during students’ clinical practica, as well as in the classroom. |  | |
| Minore (2009)  Peer-reviewed concept paper | Canada | Concept paper based on interviews, focus groups and qualitative survey data derived from seven studies  Emerging practice (level 4) | Not reported | Paraprofessional health workers  Yes | To examine the evolving role of First Nation health workers, and discuss the proposed introduction of competency-based standards for their education, certification and regulation | Professional organisations establish and uphold professional standards. | Need CHRs to be responsive to changing local circumstances. | Questions of liability => CHRs underutilised and marginalised within care team and face conflicting expectations. |  | Multi-stage consultation identified 22 core competencies for paraprofessionals within 7 domains: 1) Aboriginal and primary health care; 2) empowerment, community relations and cultural competence; 3) prevention, promotion and protection; 4) emergency care; 5) communications; 6) ethics, leadership and teamwork; and 7) administration. Need to determine scopes of practice and expected competencies for paraprofessionals, on which standards for accredited educational programs and certification can be based. |  | |
| Murray (2006)  Peer reviewed commentary paper | Aust | Commentary  Emerging practice (level 4) | Not reported | General workforce  No | To explore how the health care needs of rural, remote and Indigenous communities are to be met given future widespread health workforce shortages | Need for improved consistency in national competency standards and qualifications about where practice responsibility lies => clear job specifications and training pathways for IHWs with clinical practice roles. |  | Room for expanding scope of clinical practice e.g., nurse practitioners and flexible local medical delegation, supported by standard treatment manuals and standing orders linked to poisons legislation reform. Some IHWs provide advanced care e.g. haemodialysis, midwifery. |  | A framework for less regulated, flexible delegation of clinical duties by doctors to other members of the health team locally. With Indigenous community partnerships, regional accessibility and well-resourced programs for student support, it is possible to substantially increase the numbers of Indigenous students in professional training. Short generic training pathways could equip IHWs for expanded clinical roles under delegation. No one professional group represents “the answer”  . | . | |
| Nagel, Frendin, Bald (2009)  Discussion paper | Aust | Synthesis of clinical and academic experiences  Promising practice (level 3) | Not reported | AOD workforce  No | To explore some of the background to the alcohol and other drugs workforce, link the current direction with relevant Northern Territory experience and evidence, and present specific recommendations for the next three to five years | Level, continuity, or allocation of funding affects PHCs ability to recruit, develop, support and sustain staff members. |  | Centralised executive support appropriate for remote AOD workforce that is not highly skilled or able to self-advocate. AOD role is new and vulnerable to pressure from acute care needs and professional isolation - held together by coordinator commitment. Needs strong clinical leadership, supervision, service agreements. |  | Opportunity to develop services, overcome worldview difference, develop empowerment and ownership and promote access. Workforce support to deliver services in personal and practical ways through professional development, peer support, advocacy as a group, career structure, travel and accommodation.  Regular clinical supervision and clinical review, clarity of management accountability and professional responsibility, and clarity of role. Focus on developing identity and cohesion across the workforce as an important ingredient for success | 20 positions filled with the vast majority stayed. | |
| Nelson (2015)  Peer reviewed paper | Aust | Participatory Action Research (PAR) to elicit the reflections of five Aboriginal counsellors; thematic analysis  Emerging practice (level 4) | Yes | Indigenous mental health practitioners who undertook ten days of formal training in CBT  Yes | To identify issues affecting the clinical supervision of all Aboriginal and Torres Strait Islander mental healthcare workers, and propose alternative supervision models |  | 50% Indigenous MH professionals are remote with limited local support. Shared histories (with clients) of stressors and social determinants, community obligations. | Current practices lead to high stress and burnout. | Challenging and potentially damaging conditions; stay because duty to communities; high need for effective supervision and support not acknowledged. Generally not satisfied. | Alternative supervision models 1) cultural supervisors; 2) dual supervisors; 3) accessibility to consultation, and 4) communities of practice for remote workers through modern technologies.  Effective supervision by: 1. clinical expertise for skill acquisition and development; 2. personal support recognizing the specific issues (e.g. “the blur”) faced by Indigenous practitioners; and 3. cultural/community understanding that informs the clinical and personal support. Investment in best-practice supervision may reduce costs of cyclical workforce recruitment and unmanaged mental illness. |  | |
| Panzera (2016)  Peer-reviewed paper | Aust | Action research methodology informed by systems thinking. Four stages of workforce planning: needs assessment; health service model redesign; skills-set assessment and workforce redesign; and development of workforce and training plan.  Promising practice (level 3) | Yes | General workforce  No | To exemplify how participatory regional health workforce planning processes can accurately model current and projected local workforce requirements | The level, continuity, or allocation of resourcing affected PHCs ability to recruit, develop, support and sustain staff. |  | Speciﬁc skills shortages – e.g. IHW and a pharmacist in Yarrabah. Workforce solutions need to be cost neutral i.e. more effective deployment of staff, or skills extension. |  | Flexible health workforce roles, including delegated practice models (PAs and expanded IHW roles) and more task allocation (e.g. Nurse Practitioners and Rural and Isolated Practice Endorsed Registered Nurses) important in responsive health workforce. Bringing together healthcare providers from all disciplines and health sectors in planning health systems and workforce and training solutions based on local needs. Focus on extending competencies and skills sets. | Stronger health systems and workforce training solutions delivered e.g. task substitution and redistribution, increased delegated practice models. Stable and sustainable local workforce. | |
| Roach (2007)  Peer-reviewed paper | Aust | Data collection and analysis of government statistics and interviews with general practitioners, local managers and regional employers and organisations.  Emerging practice (level 2) | Not reported | Doctors  No | To describe the extent to which general practitioners in the Kimberley region are available for doctor-provided primary care and how primary care availability relates to need and standardised population. |  |  | Region had only half the practitioners needed. IHWs and nurses competently provide much care but need more resources - increases could have more rapid impact than long-term increase in supply of doctors. Local staff to improve continuity of care. |  | Initiatives: addressing extensive infrastructure needs, funds for salaries, promoting rural health care to all health professionals, need more IHWs, nurses, allied health professionals and doctors. HW need to be supported to undertake advanced training to better fulﬁl more complex roles (task substitution). IHWs trained for 20 years by KAMSC are element of workforce where a signiﬁcant difference could be made. |  | |
| Schmidt (2016)  Peer reviewed paper | Aust | Content analysis on 377 project records of health worker activity; interviews with 21 stakeholders. Data thematically analysed.  Promising practice (level 3) | Yes | Community health workers  Yes | To explore how a client-centred Chronic Care model was implemented by Indigenous Health Workers (IHWs) at participating sites in a trial of IHW-led case management | The level, continuity, or allocation of resourcing affected PHCs ability to recruit, develop, support and sustain staff members. | Lack of respect for the cultural role and training of the IHWs. | Barriers to performance of IHW - management and staff turnover; limited orientation of new staff; poor training; poor team communication and coordination; practices not adhering to chronic care guidelines; complex clinical information systems and poor maintenance of information technology; having to prioritise acute care demands. |  | Provision of training to support the IHW role; communication of the IHWs role to individual workers and their colleagues; IHWs knowledge of their clients and environment; and ongoing support by the ICST were enablers that increased IHWs’ confidence and capacity to provide chronic disease care and service coordination.  A greater emphasis on engaging clinical leaders and local champions about IHWs role in Chronic disease care would have improved team work. | A skilled and dedicated IHW is insufficient to improve chronic disease outcomes in the absence of a supportive service model. Barriers are systems issues and not a reflection of their competence or capacity.  High satisfaction was reported by IHWs and many clients. IHWs were most able to strengthen systems and practice, where they have skills  and knowledge i.e. client self-management support, and linking with community and other services and resources. | |
| Walker (2011)  Peer reviewed paper | Aust | Semi-structured interviews  Emerging practice (level 4) | Yes | IHW  Yes | To explore factors operating at the level of the clinic and the community that influence the development of the oral health role of Indigenous Health Workers | Government legislation and policy defines availability, capacity and support for groups of healthcare professionals. |  | Severe clinical demands on remote IHWs; separation of oral from general health; and difficulties of developing appropriate training and management. | High priority given to oral health by Indigenous Health Workers. | Sustainable role development requires development of innovative resourcing, training and management strategies to support uptake of new roles by health personnel. Need research on development and evaluation of tele-mentoring strategies using existing satellite facilities in remote communities. Strong support by IHWs to oral health role development is necessary but insufficient to facilitate oral health role development |  | |
| Watson (2013) | Aust | Qualitative focus group, thematic analysis  Emerging practice (level 4) | Yes | IHW  Yes | To identify key areas that Indigenous and non-Indigenous child health professionals working within Indigenous communities felt were important in providing support for their roles. |  | Indigenous Child Health Workers in Australia play the role of cultural brokerage in their communities. | ICHWs signiﬁcantly contribute to acceptability, access and use of health services. Conﬁdence important for team collaboration. | Participants conﬁdent in knowledge about infant care and illness management. | Role support for ICHW and CHW included being treated as equal team members in a culturally respectful manner and having identiﬁed opportunities for multidisciplinary communication and collaboration. Need clear role responsibilities, supported learning and suitable resources for role performance. CHW require cultural awareness and capability, resource provision, educational opportunities, collaboration with colleagues and peers, and professional mentorship. Recognition of, and consideration for, centrality of the IHW role was important for care provision and IHW conﬁdence. Cultural appropriateness important for interaction amongst colleagues and educational resources. |  | |
| Weymouth (2007)  Peer reviewed paper | Aust | Mixed method design – surveys and interviews thematically analysed  Promising practice (level 3) | Yes | Nurses  No | To examine the practice of distance management to gain a better understanding on the retention of registered nurses |  | Major barriers to distance management of Australian nurses were distance and remoteness itself. | Lack of management support increased frustration and stress, and led to staff turnover. Frequent changes of remote managers. Important management practices: recruitment and orientation, occupational health and safety, responsiveness, staff leave and replacement, professional development and review, exit interviews. | Excessive mental and physical stress led to burn-out - accepted as normal work culture. A third of RNs said support by management after a critical incident was poor. Professional support by the Bush Crisis Line was highly regarded. | Distance management by knowing context, communication and interpersonal skills, team roles clearly delineated and communicated. Availability of staff development, leave, relief staff, professional feedback, debriefing, support and conditions of service.  Recommendations:  Recruitment: timely and proactive.  Orientation: to practice, the service and community.  Valuing RANs: include in mentoring, policy development, review, decision making and quality improvement, facilitating work exchanges.  Retention Incentives: study assistance and practical incentives.  Staff travel: subsidised.  Leave management: planned leave and suitable locum relief staff.  Professional development and career structure: fast-track qualifications in remote health.  Infrastructure: assess and implement repair and maintenance  Occupational Health and Safety: prompt, effective appropriate standardised response, investigation and follow-up to critical incidents.  Violence: proactive strategy and monitoring and response systems.  Burn-out: CRANA Bush Crises Lines guidelines.  Improved Communication: RANs “heard” by management team.  Exit Interviews: with all staff.  Support Resources: to improve equity between professionals | Respondents expressed satisfaction with roles, but dissatisfaction with infrastructure, support and management. | |
| Williams (2003)  Peer reviewed article | Aust | Interviews with PHC workers  Emerging practice (level 4) | Not reported | IHW  Yes | To examine the possible OHS costs from the complex use of emotions and Aboriginal identity for Aboriginal primary health care workers while successfully carrying out a human service health job. |  |  |  | IHWs (particularly women) had worst emotional exhaustion after Indigenous managers. Emotional exhaustion is first stage of burnout and can precede physical ill-health. Situation for IHW more serious because often interacting with pre-existing physical ill-health. | Preventing burnout by using concepts of emotional labour (linked to values of care, reciprocity and respect, and obligations to carry out cultural practices in terms of Aboriginal identity such as attendance at funerals) and obligatory community labour (does not fit into western concepts of "work", "non-work" and "voluntary work”). |  | |
| Wilson (2015)  Peer reviewed paper | Aust | Qualitative interviews and thematic analysis.  Emerging practice (level 4) | Yes | General workforce  No | To explore the attitudes and characteristics of non-Aboriginal health professionals working in Aboriginal health. |  |  |  | Attitudes of non-Indigenous professionals: practical knowledge, fear, difficulty; and learning to practice regardless. | Non-Aboriginal health professionals can use the groupings to reflect on levels of confidence, attitudes, characteristics, experiences, approaches and assumptions to Aboriginal health, as an important precursor to practice in Aboriginal health. The groupings can also enable group discussions about working together in Aboriginal health.  Characteristics that assist include an awareness of cultural identity, reflection on one’s own position and awareness of Aboriginal history. These are relevant for professional development, training and education at university level, and health service delivery. Support networks and mentoring could assist. |  | |
| Zhao (2017)  Peer reviewed paper | Aust | Dynamic regression analysis of NT Department of Health payroll and financial data for the health workforce in 54 remote clinics, 2004–2015.  Promising practice (level 3) | Yes | General workforce  No | To describe temporal changes in workforce supply in government-operated clinics in remote NT communities through a period in which there has been a substantial increase in health funding | Level, continuity or allocation of funding affected PHCs ability to recruit, develop, support and sustain staff. |  | Most remote NT PHCs were unable to sustain workforce but had a heavy reliance on short-term agency employed nurses. |  | Invest in developing stronger career pathways for AHPs and nurses and implementing more robust health service models that better support the supply and retention of long term clinical staff. | Despite substantial increases in resourcing, an imperative remains to implement more robust health service models that better support the supply and retention of resident health staff. | |
