# Supplementary material for: Working well: a systematic scoping review of the Indigenous primary healthcare workforce development literature
Source: BMC Health Serv Res. 2019 Oct 29;19:767. doi: 10.1186/s12913-019-4580-5 (PMC6819619; doi:10.1186/s12913-019-4580-5)
Supplement: Supplementary file 2 — Additional file 2. Medline search 1. An example search to demonstrate the search strategy. [file 12913_2019_4580_MOESM2_ESM.docx]

**Medline search 1**

The Medline search is reproduced in full below.

Medline search 3/1/2018

# Search Results

1 indigenous.mp. or exp Oceanic Ancestry Group/ 36353

2 aborigin*.mp. 9347

3 torres strait island*.mp. 1249

4 exp Indians, North American/ or "native american*".mp. 16996

5 inuit.mp. or exp Inuits/ 4989

6 maori.mp. 2898

7 "first nation*".mp. 4153

8 metis.mp. 339

9 "american Indian*".mp. 6235

10 "Native Hawaiian".mp. 728

11 "Tangata Whenua".mp. 7

12 native alaskan.mp. 61

13 iwi.mp. 84

14 Tangata Whenua.mp. 7

15 tribal.mp. 3837

16 or/1-15 68061

17 healthcare.mp. 191459

18 (health adj1 care).mp. [mp=title, abstract, original title, name of substance word, subject heading word, keyword heading word, protocol supplementary concept word, rare disease supplementary concept word, unique identifier, synonyms] 743561

19 primary health care.mp. or exp Primary Health Care/ 157251

20 health.mp. or exp Health/ 2674654

21 or/17-20 2775485

22 service.mp. 332730

23 provider.mp. 50854

24 program.mp. 468766

25 (clinic or center or centre).mp. 737283

26 exp "Delivery of Health Care"/ 1025644

27 exp Health Services, Indigenous/ 2895

28 exp Health Services Accessibility/ 105144

29 community program.mp. 422

30 exp Health Services Research/ 161045

31 or/22-30 2308779

32 workforce.mp. 19974

33 retention.mp. 177279

34 recruitment.mp. 119705

35 coordination.mp. 100283

36 develop*.mp. 4226666

37 exp Personnel Selection/ 12466

38 exp Health Manpower/ 12556

39 exp Staff Development/ 9050

40 exp Professional Role/ 80622

41 exp Personnel Management/ 145424

42 or/32-41 4729131

43 (model* or process* or strateg* or plan*).mp. 7013441

44 exp Practice Guidelines as Topic/ 112607

45 exp "Quality of Health Care"/ 6690782

46 exp Cultural Competency/ 4731

47 exp Capacity Building/ 1851

48 exp Professional Competence/ 109934

49 exp Health Planning/ 338541

50 exp Models, Organizational/ 19100

51 or/43-50 11938142

52 42 or 51 13939908

53 Australia.mp. or exp Australia/ 166385

54 New Zealand.mp. or exp New Zealand/ 67375

55 Canada.mp. or exp Canada/ 185847

56 exp United States/ or USA.mp. 1394904

57 or/53-56 1767019

58 16 and 21 and 31 and 52 and 57 8419

59 limit 58 to (english language and humans and yr="2000 -Current") 6748

60 limit 59 to (congresses or editorial or letter or news or patient education handout) 264

61 59 not 60 6484

62 limit 61 to yr="2000 - 2010" 3259

63 remove duplicates from 62 3013

64 limit 61 to yr="2011 -Current" 3225

65 remove

**Medline search 2**

Supplementary search of Medline

The search was conducted on 31 March, 2018 in Medline (including Epub Ahead of Print, In-Process & Other Non-Indexed Citations) / Ovid.

Search strategy

The database was searched with the terms below and their corresponding MeSH terms:

1. Indigenous OR Aborigin* OR “Torres Strait Island”* OR Inuit OR Maori OR Iwi OR Tangata Whenua OR “First Nation”* OR Metis OR “Native American”* OR “American Indian”* OR “Native Hawaiian” OR “Native Alaskan”* OR tribal

2. Healthcare OR health care OR primary health care OR health

3. Workforce OR worker OR recruitment OR employee OR health manpower OR health personnel

4. Motivation OR productivity OR performance OR satisfaction OR incentive OR quality of health care OR professional competence

5. Australia OR Canada OR USA OR New Zealand

6. AND/1-5

The results were limited to studies published from2000 to the present (2018) in English.

After results were checked for duplicates, the search retrieved 826 studies for review.

The Medline search is reproduced in full below.

Medline search 31/3/2018

# Search Results

1 indigenous.mp. or exp Oceanic Ancestry Group/ 33983

2 aborigin*.mp. 8872

3 torres strait island*.mp. 1211

4 exp Indians, North American/ or "native american*".mp. 15758

5 inuit.mp. or exp Inuits/ 4409

6 maori.mp. 2781

7 "first nation*".mp. 3851

8 metis.mp. 314

9 "american Indian*".mp. 5815

10 "Native Hawaiian".mp. 674

11 "Tangata Whenua".mp. 5

12 iwi.mp. 80

13 Native Alaskan*.mp. 87

14 tribal.mp. 3580

15 or/1-14 63516

16 healthcare.mp. 180450

17 (health adj1 care).mp. [mp=title, abstract, original title, name of substance word, subject heading word, keyword heading word, protocol supplementary concept word, rare disease supplementary concept word, unique identifier, synonyms] 696302

18 primary health care.mp. or exp Primary Health Care/ 147599

19 health.mp. or exp Health/ 2508780

20 or/16-19 2604326

21 workforce.mp. 19242

22 exp Health Manpower/ 12107

23 health personnel.mp. or exp Health Personnel/ 517788

24 worker.mp. or exp COMMUNITY HEALTH WORKERS/ 29635

25 employee.mp. 30542

26 or/21-25 581223

27 motivation.mp. or exp MOTIVATION/ 189007

28 productivity.mp. or exp Efficiency/ 74815

29 performance.mp. or exp "TASK PERFORMANCE AND ANALYSIS"/ or exp WORK PERFORMANCE/ or exp EMPLOYEE PERFORMANCE APPRAISAL/ 831754

30 satisfaction.mp. or exp Personal Satisfaction/ 174223

31 incentive.mp. 16654

32 exp "Quality of Health Care"/ 6111448

33 exp Professional Competence/ 103808

34 or/27-33 6837583

35 Australia.mp. or exp Australia/ 157360

36 New Zealand.mp. or exp New Zealand/ 63843

37 Canada.mp. or exp Canada/ 173485

38 exp United States/ or USA.mp. 1319150

39 or/35-38 1669693

40 15 and 20 and 26 and 34 and 39 1001

41 limit 40 to (english language and humans and yr="2000 -Current") 840

42 limit 41 to (editorial or letter) 14

43 41 not 42 826

44 remove duplicates from 43 826
